# Inpatients with a history of suicide attempts in routine clinical care exhibit specific characteristics regarding sociodemographics, life events, personality, and symptom burden

**DOI:** 10.1038/s41598-024-66987-x

**Published:** 2024-07-31

**Authors:** Theresa J. Gemke, Rüdiger Zwerenz, Manfred E. Beutel, Matthias Michal, Jörg Wiltink, Mareike Ernst

**Affiliations:** 1 Department of Psychosomatic Medicine and Psychotherapy, University Medical Center of the Johannes Gutenberg-University Mainz, Mainz, Germany; 2 Department of Clinical Psychology, Psychoanalysis and Psychotherapy, Institute of Psychology, University of Klagenfurt, Klagenfurt am Wörthersee, Austria

**Keywords:** Inpatients, Day clinic, Psychosomatic, Psychotherapy, Suicide attempts, Risk factors, Risk factors, Medical research, Signs and symptoms

## Abstract

Research indicates that patients with a lifetime history of suicide attempts are particularly burdened. However, investigations of their characteristics and comparisons with other patients are scarce. This study aimed to fill this research gap, using routine clinical data and guided by theoretical models. Data of *N* = 706 patients (54.4% women) was collected at the psychosomatic inpatient/day-clinic unit of a German university clinic. It comprised sociodemographic data and information about previous experiences (e.g., childhood abuse and neglect), symptom measures (e.g., the PHQ-9) and individual differences (e.g., the level of personality functioning assessed with the OPD-SQS). Groups were compared using independent t-tests or χ^2^-tests. Of the total sample, 118 patients (16.7%) reported suicide attempts. Those with a history of suicide attempts were more likely to have a migration background and a lower level of education, smoke (heavily) and use illegal substances. They reported lower levels of personality functioning, more current symptoms and traumatic previous experiences of abuse and neglect. Screening for previous suicidal behavior as well as associated factors can yield valuable information for clinical practice. Many group differences map onto previously observed specific risk factors for suicidal behavior, supporting the conceptual models and underscoring their relevance among clinical populations as well.

## Introduction

Suicidal thoughts and behavior (STBs) constitute a global mental health problem. While the point prevalence of suicidal thoughts is around 8% in representative German population samples^1^, suicidal behavior is a rather rare occurrence with just under 10,000 suicide deaths per year in all of Germany^2^ and 1.9% of the population reporting previous attempts^3^. Experts point out that for every suicide death among adolescents^4^ and adults^5^, there are considerably more nonfatal attempts (by up to a factor of twenty)^6^.

Not surprisingly, the prevalence rates of both suicidal ideation and attempts were notably higher in clinical samples, with up to 50.4% of inpatients and 11% of outpatients reporting current suicidal ideation^7,8^ and 6% of outpatients seeking psychotherapy^8^ and 19.4% of chronically depressed patients included in a psychotherapy trial^9^ reporting past attempts.

In previous studies, individuals with a history of suicide attempts have been identified as a vulnerable group that differed from others in important ways, even within clinical populations. For instance, in the large STAR*D (Sequenced Treatment Alternatives to Relieve Depression) trial, they reported more comorbidities, younger age of onset of depression, and more depressive episodes than other depressed patients. They were also more likely to experience current suicidal ideation^10^. Moreover, in a study that compared people with alcohol dependence who had attempted suicide with those who had not, those reporting suicide attempts were found to have a significantly more severe alcohol addiction. They were also more likely to be dependent on other drugs and had a higher prevalence of mental disorders such as depression, mania, panic disorder and social phobia^11^. These characteristics indicate a greater severity of mental distress and have important implications for treatment planning.

The distinction between suicidal ideation and *behavior* is a critical frontier in suicide research^12^, as the two are distinct entities shaped by specific risk and protective factors. This notion finds expression in the modern models of the ideation-to-action framework^13^. As such, the present work is guided by the Integrated Motivational-Volitional Model (IMV) of Suicidal Behavior^14^. Within the IMV Model, factors that influence both the emergence and the maintenance of suicidal ideation within the motivational phase are called threat-to-self- (TSM) and motivational moderators (MM). For instance, self-criticism was positively associated with higher levels of patients’ depressive symptoms^15^, which suicidal ideation is conceptualized to be a part of. Moreover, the IMV model posits that those with a history of suicide attempts will also exhibit higher levels of these motivational phase variables^14^. Among students who had attempted suicide, self-critical individuals also reported greater intent to die and chose more lethal methods^16^. The factors that are assumed to differentiate most clearly between those who engage in suicidal behavior and those who do not are called volitional moderators (VM). They comprise variables relating to the construct of acquired capability for suicide (i.e., desensitization to pain and fearlessness of death)^14^. For instance, in a representative population survey, suicide attempt survivors reported having suffered more childhood physical and sexual abuse than others^9^.

More knowledge about the characteristics of those at risk for suicidal behavior can help clinicians to customize their treatment according to these patients’ unique challenges and needs. However, comprehensive investigations of patient samples are scarce and most of the presented evidence regarding suicidal ideation and behavior came from selective trials instead of naturalistic settings or routine clinical care. In addition, most studies compared specific (diagnosis) groups, e.g., patients with depression or alcohol dependence. Therefore, the question remains to what extent these study results can be applied to naturalistic patient samples with a variety of diagnoses.

The present work sought to fill this research gap. In particular, we were interested in finding out which proportion of individuals seeking inpatient/day-clinic treatment at a psychosomatic clinic in Germany reported a history of suicide attempts. Going from there, we aimed to compare them to the other patients regarding their sociodemographic characteristics, symptom burden, and proposed specific risk factors for suicidal behavior. Drawing on the IMV model as a conceptual framework, our main focus was on moderating variables: we expected an increased prevalence/greater expression of volitional moderators (including the experience of child maltreatment and personality pathology) among those with a history of suicide attempts compared to those without. We also expected threat-to-self moderators (such as self-criticism and coping) to differ between the groups in the sense that those reporting previous suicide attempts are more vulnerable, however, these differences should have smaller effect sizes than those of volitional moderators. Further comparisons between the groups regarding sociodemographic characteristics were conducted in an exploratory manner.

## Methods

### Participants and setting

We used routine clinical data from inpatients and patients from the day clinic who were treated at a Clinic for Psychosomatic Medicine and Psychotherapy at a German university hospital. Data was collected from 2018 to 2021. We used all cases for whom information about previous suicide attempts was available and did not apply any in-/exclusion criteria. The primary aim of this data is to evaluate the clinic's services in the sense of quality control. Its use for research purposes is regulated by the State Hospital Act and was approved by the Rhineland-Palatinate Chamber of Physicians (Nr. 837.191.16 (10510)). All patients provided informed consent. All assessments were performed in accordance with the relevant guidelines and regulations.

Psychosomatic medicine includes the treatment of the entire spectrum of mental disorders, except for acute psychotic, severe brain-organic and severe drug-related disorders. Especially affective, somatoform and eating disorders as well as trauma sequelae and somato-psychic disorders (e.g., cancer or heart disease) with high complexity and chronicity are treated in the psychosomatic day clinic or as inpatients by applying a multimodal therapy approach. A day clinic treatment concept (with 8 h/weekday, before and after which patients are at home) is aimed at patients who have not yet received sufficient support through exclusively outpatient treatment or who are unable to take advantage of full inpatient treatment (e.g., due to responsibilities at home). For more information about the patients treated in this setting in Germany, see Doering, Herpertz^17^.

### Data collection

Data were collected via self-report questionnaires. In terms of sociodemographic characteristics, patients reported their sex/gender, age, level of education, marital status, living situation and migration background (operationalized in line with the German microcensus^18^, i.e., if they or at least one of their parents do not have German citizenship by birth). As the information about cigarette smoking, migration background and the Childhood Trauma Screener, which is described below, were added to the routine monitoring during the data collection evaluated, there is a certain proportion of missing data on these variables. In addition, regarding their health behavior, patients reported whether they smoked (response format: never; former smoker; occasionally, not daily; fewer than 5 cigarettes/day; more than 5 cigarettes/day), their usual consumption of alcohol (drinks per day) and illegal drugs (yes/no). Moreover, patients were asked whether they had ever attempted suicide.

Validated questionnaires were used to capture current symptom burden as well as relevant life events and individual differences.

The depression module of the Patient Health Questionnaire (PHQ-9) was used to assess current depression symptoms^19,20^. This widely used screening measure consists of nine questions that are based on the diagnostic criteria for major depressive disorder outlined in the Diagnostic and Statistical Manual of Mental Disorders (DSM-5). The items cover several domains of depressive symptoms, including mood, sleep, appetite, energy levels, concentration, and suicidal ideation. Participants rate the frequency of each symptom over the past two weeks from "0 = not at all" to "3 = nearly every day". Its total score thus ranges from 0 to 27. In previous investigations of clinical samples, its internal consistency was good (ω = 0.85)^21^. As part of the present investigation, we report both the sum score and the suicidal ideation item (item 9) which we analyzed separately and coded in a binary fashion in line with previous work (e.g.^22^); distinguishing between those reporting no suicidal ideation at all (response option 0) and those reporting suicidal ideation irrespective of its frequency (combining response options 1, 2, and 3).

Patients completed the Childhood Trauma Screener (CTS), a short version of the established Childhood Trauma Questionnaire, which contains five items capturing potentially traumatic childhood experiences (emotional, physical and sexual abuse; emotional and physical neglect)^23,24^. For each of these five items, patients rate the commonness of occurrence whilst growing up from “0 = not at all” to “4 = very often”. We used a cut-off validated in representative population samples for each item to identify a significant occurrence of the respective type of abuse/neglect. The validated cut-offs were “2 = several times” for physical and emotional abuse, “1 = rarely” for sexual abuse, and “3 = often” for physical and emotional neglect^23^.

The Brief Resilient Coping Scale (BRCS) was used to assess the psychological resilience of patients in stressful situations^25,26^. This questionnaire contains four items, asking participants to evaluate their behavioral patterns in certain situations, e.g., "I actively look for ways to replace the losses I encounter in life". Patients rate the items regarding their accuracy from “1 = does not describe me at all” to “5 = describes me very well”. Sum values range between 4 and 20, with higher values indicating more resilient coping. In previous studies, its internal consistency was adequate (α = 0.78)^25^.

The OPD Structure Questionnaire (OPD-SQS) was employed to assess the level of personality functioning, a dimensional approach to personality pathology^27^. Its twelve items are summarized in three correlated subscales: self-perception, shaping contact and relationship model. Participants are asked to rate each statement (e.g., "I sometimes experience myself as a stranger") from “0 = fully disagree” to “4 = fully agree”. The total score can thus vary from 0 to 48, with higher values indicating more severe deficits. Previous studies in clinical samples (α = 0.89)^28^ and based on representative population surveys^27^ have attested to its good psychometric properties.

The Depressive Experiences Questionnaire Self-Criticism 4 (DEQ-SC4) was utilized to measure Self-criticism in patients^29^. Patients were asked to evaluate four items, e.g., "I tend to be very critical of myself". The possible answers ranged from “1 = does not apply to me at all” to “7 = fully applies to me” and the total score ranges from 4 to 28. Higher values indicate more self-criticism. Internal consistency was good with α = 0.75^29^.

### Statistical analysis

Descriptive statistics are reported as absolute numbers and percentages or as mean values and standard deviations, respectively. We also report effect sizes to aid in the judgement of the relevance of the observed differences. They are interpreted following Cohen^30^.

## Results

### Sample characteristics

The analysis sample comprised 706 individuals for whom data about previous suicide attempts was available. Their age ranged from 17 to 77 years, with a mean age of 37.77 years (*SD* = 14.65). Of this sample, 54.4% were women. The majority (*N* = 577, 81.7%) had a primary or secondary diagnosis of depression. Detailed patient characteristics are reported in Table 1.

**Table 1:** Sample characteristics (*N* = 706).

	*N* (%)/*M* (*SD*)
Sociodemographic characteristics	
Sex/gender (women)	384 (54.4)
Age	37.77 (14.65)
Migration background^a^	118 (31.2)
Partnership	357 (50.6)
Married	189 (26.8)
Parenthood	248 (35.1)
Level of education (Abitur)	397 (56.2)
Main diagnoses	
Primary or secondary diagnosis of depression	577 (81.7)
F32.0	4 (0.6)
F32.1	120 (17.0)
F32.2	41 (5.8)
F32.9	45 (6.4)
F33.0	11 (1.6)
F33.1	250 (35.4)
F33.2	84 (11.9)
F34.1	22 (3.1)
Primary or secondary diagnosis of anxiety	222 (31.4)
F40.-	50 (7.1)
F41.-	172 (28.7)
Life events^a^	
Childhood trauma screener (CTS) (sum score)	4.84 (3.98)
Prevalence of emotional abuse	127 (38.4)
Prevalence of physical abuse	69 (20.8)
Prevalence of sexual abuse	62 (18.7)
Prevalence of emotional neglect	95 (28.7)
Prevalence of physical neglect	51 (15.4)
Personality and individual differences	
Resilient coping (BRCS sum score)	12.59 (3.36)
Personality functioning (OPD-SQS sum score)	25.23 (9.49)
Self-Criticism (DEQ-SC4 sum score)	20.40 (5.59)
Use of harmful legal and illegal substances	
Never	175 (48.7)
Former smoker	68 (18.9)
Occasionally, not daily	17 (4.7)
Fewer than 5 cigarettes per day	16 (4.5)
More than 5 cigarettes per day	77 (21.4)
Alcohol consumption	1.14 (1.05)
Use of illegal substances	42 (5.9)
Mental distress, suicidal thoughts and behaviors	
Depression symptoms (PHQ-9 sum score)	15.05 (5.55)
Current suicidal ideation (PHQ-9 item)	348 (49.3)
Reported previous suicide attempt(s)	118 (16.7)

^a^As data on cigarette smoking, migration background and the CTS were only available for a proportion of the sample, the respective percentages refer to the patient sample for whom this data was available: *N* = 359 in the case of cigarette smoking, *N* = 378 in the case of migration background; *N* = 331 in the case of the CTS (sum).

### Group comparisons

In total, 118 patients (16.7% of the total sample) reported previous suicide attempts. Among them were 73 women (61.9% of those reporting attempts) and 45 men (38.1% of those reporting attempts), so there was no significant gender difference (*p* = 0.085). Patients who reported suicide attempts did not differ significantly from others regarding their age (*p* = 0.40), the presence of a stable partnership (*p* = 0.31), their marital status (*p* = 0.09) or in terms of parenthood (*p* = 0.49). However, patients with a migration background (χ^2^ (1, *N* = 378) = 5.68, *p* = 0.024, φ = 0.12) and a low level of education (χ^2^ (1, *N* = 689) = 15.07, *p* < 0.001, φ = 0.15) were overrepresented among those reporting suicide attempts. Moreover, patients who smoked (heavily) (χ^2^ (1, *N* = 358) = 33.94, *p* = 0.001, φ = 0.31) and used illegal substances (χ^2^ (1, *N* = 696) = 11.68, *p* = 0.002, φ = 0.13) were more likely to have a history of suicide attempts. There was no significant difference regarding alcohol consumption (*p* = 0.24).

We further compared patients with and without lifetime suicide attempts regarding their symptom burden including current suicidal ideation. Before the start of treatment, patients with a history of suicide attempts reported significantly more depression symptoms on the PHQ-9 (*M* = 17.17, *SD* = 5.15 vs. *M* = 14.63, *SD* = 5.54; *t*(672) = 4.45, *p* < 0.001, *d* = 0.46). A greater proportion of them also reported current suicidal ideation (χ^2^ (1, *N* = 699) = 38.83, *p* < 0.001, φ = 0.24).

**Figure 1:**
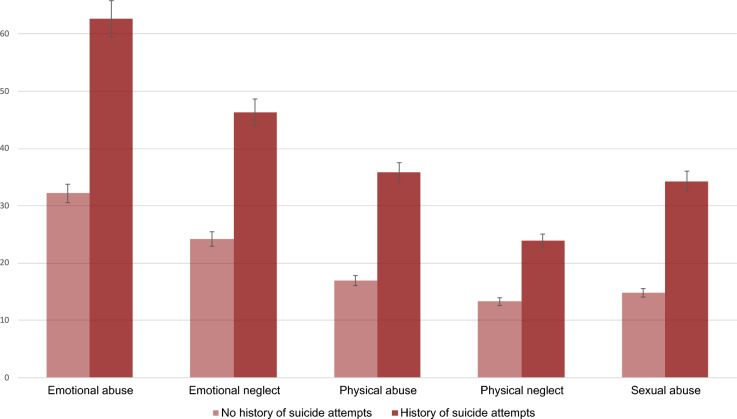
Prevalence rates of the different types of childhood abuse and neglect assessed by the Childhood Trauma Screener (CTS) among inpatients/day-clinic patients with and without suicide attempts. While individuals with a history of suicide attempts reported more experiences of all domains of the CTS, the largest difference pertained to emotional abuse (followed by sexual abuse).

Patients with a history of suicide attempts reported more instances of childhood abuse and neglect, as indicated by the CTS sum score (*M* = 7.49, *SD* = 4.58 vs. *M* = 4.17, *SD* = 3.51; *t*(329) = 6.47, *p* < 0.001, *d* = 0.89) and the elevated prevalence rates of all types of abuse and neglect (see Table 2), with the most notable differences observed for the prevalence of emotional abuse (62.7% among those with a history of suicide attempts vs. 32.2% among those without). Figure 1 yields an additional visualization of the mean values.

**Table 2:** Comparison of patients with and without a history of suicide attempts.

	Patients with a history of suicide attempts (*N* = 118)	Patients without a history of suicide attempts (*N* = 588)	Significance level	Test value	Phi/Cohen’s d
	*N* (*%*)/*M *(*SD*)	*N *(*%*)/*M *(*SD*)	*P*	*t *(*df*)	*φ*/*d*
Sociodemographic characteristics					
Sex/gender (women)	73 (61.9)	311 (52.9)	0.085	–	–
Age	36.74 (14.99)	37.97 (14.58)	0.40	–	–
Migration background^a^	24 (45.3)	94 (28.9)	**0.024**	5.68 (1)	0.12
Partnership	55 (46.6)	302 (51.4)	0.31	–	–
Married	24 (20.3)	165 (28.1)	0.09	–	–
Parenthood	38 (32.2)	210 (35.7)	0.49	–	–
Level of education (Abitur)	48 (40.7)	349 (59.4)	** < 0.001**	15.07	.15
Life events					
Childhood trauma screener (CTS) (sum score)^a^	7.49 (4.58)	4.17 (3.51)	** < 0.001**	6.47 (329)	0.89
Prevalence of emotional abuse	42 (62.7)	85 (32.2)	** < 0.001**	21.06 (1)	0.25
Prevalence of emotional neglect	31 (46.3)	64 (24.2)	** < 0.001**	12.27 (1)	0.19
Prevalence of physical abuse	24 (35.8)	45 (17.0)	** < 0.001**	11.45 (1)	0.18
Prevalence of physical neglect	16 (23.9)	35 (13.3)	**0.038**	4.66 (1)	0.12
Prevalence of sexual abuse	23 (34.3)	39 (14.8)	** < 0.001**	14.26 (1)	0.21
Personality and individual differences					
Resilient coping (BRCS sum score)	12.56 (3.54)	12.59 (3.32)	0.92	–	–
Personality functioning (OPD-SQS sum score)	29.53 (8.92)	24.35 (9.36)	** < 0.001**	5.45 (679)	0.56
OPD self-perception	9.06 (4.02)	7.63 (4.14)	**0.001**	3.43 (694)	0.35
OPD shaping contact	9.50 (3.39)	8.25 (3.58)	** < 0.001**	3.47 (692)	0.35
OPD relationship model	10.88 (3.73)	8.45 (4.06)	** < 0.001**	5.93 (691)	0.61
Self-criticism (DEQ-SC4 sum score)	20.88 (5.31)	20.31 (5.64)	0.38	–	–
Use of harmful legal and illegal substances					
Cigarette smoking^a^			** < 0.001**	33.94	0.31
Never	11 (9.3)	164 (27.9)			
Former smoker	5 (4.2)	63 (10.7)			
Occasionally, not daily	3 (2.5)	14 (2.4)			
Fewer than 5 cigarettes per day	5 (4.2)	11 (1.9)			
More than 5 cigarettes per day	23 (19.5)	54 (9.2)			
Alcohol consumption	1.31 (1.54)	1.11 (0.94)	0.24	–	–
Use of illegal substances	15 (12.7)	27 (4.6)	**0.002**	11.68 (1)	0.13
Mental distress and suicidal ideation					
Depression symptoms (PHQ-9 sum score)	17.17 (5.15)	14.63 (5.54)	** < 0.001**	4.45 (672)	0.46
Current suicidal ideation (PHQ-9 item)	89 (75.4)	259 (44.0)	** < 0.001**	38.83 (1)	0.24

Statistically significant differences are printed in bold.

^a^ As data on cigarette smoking, migration background and the CTS were only available for a proportion of the sample, the respective percentages refer to the patient sample for whom this data was available: *N* = 359 in the case of cigarette smoking, *N* = 378 in the case of migration background; *N* = 331 in the case of the CTS (sum).

We also examined whether patients with and without a history of suicide attempts differ regarding their resilience, personality functioning and self-criticism. There was no significant difference between the two groups regarding the patients' sum score in the BRCS (*p* = 0.92) or the DEQ-SC4 sum score (*p* = 0.38). However, patients with a history of suicide attempts showed higher sum scores in the OPD Structure Questionnaire (OPD-SQS) (*M* = 29.53, *SD* = 8.92 vs. *M* = 24.35, *SD* = 9.36; *t*(679) = 5.45, *p* < 0.001, *d* = 0.56), indicating more severe deficits in personality functioning. These differences also pertained to all three subscales.

## Discussion

This work aimed to collect more information about patients with a lifetime history of suicide attempts and thus to be able to better assess and target specific challenges in the everyday treatment of these patients. As previous studies on this topic were mostly based on selective trials instead of data from routine care, we were interested in which proportion of psychosomatic inpatients/day-clinic patients in routine care reported a history of suicide attempts and to which extent results from selective trials or population samples can be compared to a naturalistic setting like this.

In this study, patients who reported suicide attempts did not differ from others in all the variables of interest. However, there were some relevant differences observed in each category of investigated factors: sociodemographic characteristics, symptom burden, life events and individual differences. It is conceivable that some of these differences conferred additional vulnerability.

First, our results support the relevance of socio-demographic characteristics: consistent with an earlier, representative study from Germany^31^, the patients in our sample who had a migration background were more likely to have a history of suicide attempts. Moreover, our findings aligned with the results from population samples regarding the protective effect of higher educational attainment^32^. Both a migration background and a lower level of education could be rather distal risk factors for suicidal behavior, in the sense that they contribute to the background in which distress, and then more specifically, suicidal thoughts and actions unfold. In the context of the IMV model, this background is called the pre-motivational phase^14^ and it refers to established diathesis-stress-models^33^. Of course, a lower educational level and a migration background are unspecific influences as there are many individuals to whom these characteristics apply who never become suicidal. While the present study does not allow for causal inferences, its results point to the importance of considering the socio-ecological context. As such, most of the present findings might be classified as *risk indicators* or *correlates*, also because the study design does not allow for testing *causal risk factors*^34,35^. Still, they yield relevant information for clinical practice as they point towards factors which need to be explored in more depth. Taking the example of fewer years of education and migration background, it is crucial to ascertain what these variables mean with regard to the person's life story and current situation: for one, a lower level of education likely translates into lower wages and a higher risk of financial insecurity and living in poverty. Lower socioeconomic position was independently related to STBs in previous research^36,37^. Also, both lower socioeconomic status^38,39^ and experiences of flight and migration^40^ can implicate limited social participation and feelings of loneliness. Loneliness, in turn, is a prospective risk factor for suicidal ideation and behavior^41^. Furthermore, the individual biographies behind these factors also deserve attention, for instance, for those who migrated, was it their (or their parents') own, free decision, or did they suffer forced displacement and perhaps a traumatic flight experience? What are the more proximal stressors arising from this experience—aspects related to the migration itself, or problems in daily life having to do with, for instance, language barriers or experiences of (racial) discrimination? Answering these questions will help determine the more proximal foci that must be addressed to foster well-being and lower suicide risk (e.g.^42^).

Other differences between those with a history of suicide attempts and those without related more directly to the constructs included in the IMV model that were previously established as specific risk factors of suicidal behavior (i.e., volitional moderators): although psychosomatic inpatients and day-clinic patients, in particular, are likely to struggle with unsafe levels of alcohol consumption and use of other substances^43–45^, we found that patients with a suicide attempt in the past were more likely to smoke heavily and to use illegal drugs than others (although they did not differ significantly from other patients regarding their daily alcohol consumption). Drug use disorder also differentiated individuals with suicidal ideation from those with suicidal behavior in a large meta-analysis^46^. This finding can have different meanings: it could indicate lower risk aversion among those with a history of suicide attempts, a greater need to escape distressing subjective experiences, and/or easier availability of illegal substances through the social circles in which they move. Therefore, the use of illegal substances can confer multiple risks (besides the direct health risks). Both the use of illegal substances as well as alcohol is deemed a prospective risk factor for suicidal behavior^47,48^ because of the danger of disinhibition (in the sense of a volitional moderator within the IMV model), so that suicidal desire is more likely to become suicidal action. Along these lines, it is important to reiterate that this present study was not prospective, but a comparison based on previous attempts and current behavior. Therefore, the present results suggest the use of legal and illegal substances to be part of a temporally stable risk profile.

Furthermore, the findings of this study underline the relevance of traumatic interpersonal experiences while growing up for the emergence of suicidal behavior. All types of abuse and neglect were more common than in representative population samples^24^, mirroring previous findings^21,49^. In particular, reports of emotional and sexual abuse were substantially more common. It is thus especially striking that even among psychosomatic inpatients and day-clinic patients, a group in which so many are affected by childhood trauma, those with a history of suicide attempts exhibited substantially higher prevalence rates still, especially of emotional and sexual abuse. Their relevance for self-harm has previously been shown in other contexts^50^, including clinical samples^9,51,52^, albeit it was discussed that their effects on self-harm over the life span are likely mediated by different processes^3^.

Lastly, personality variables that were previously conceptualized as motivational moderators or threat-to-self moderators—that is, risk factors for suicidal thoughts rather than behaviors—such as resilience did *not* differ significantly between patients with and without a history of suicide attempts. The only exception was the level of personality functioning, which was substantially lower among patients who had previously attempted suicide. As a dimensional approach to personality pathology, it is in line with the new conceptions of personality disorders now part of the DSM-5 (e.g.^53^) and ICD-11^54^ that focus on difficulties relating to the self and others (including the accurate and differentiated perception and regulation of emotions as well as of interpersonal relationships), however, there is a paucity of previous research using this approach in suicide research. Evidence from population samples supports personality pathology as a mediating variable of the effects of childhood trauma^3^, mirroring the findings of a current systematic review (employing a categorical approach)^55^.

In summary, these findings have several implications for clinical practice. On the one hand, the present work expands the knowledge about the characteristics of patients with a history of suicide attempts that are not modifiable (such as migration background and low level of education) but highlight potential stressors that should be further explored in the anamnesis and treatment, especially when they cumulate. It is advisable to include such variables in future data collection and risk assessment efforts, and to investigate the ways in which they contribute to individual distress and risk (i.e., whether they constitute mere correlates or whether they comprise causal risk factors). On the other hand, some of the differences between patients with and without a history of suicide attempts pertain to characteristics that are malleable over time and that could constitute important targets for interventions, for example, the level of personality functioning was shown to improve over the course of psychotherapy^56,57^. Other differences point to the need for specialized interventions and assistance to mitigate future risks, such as the use of harmful substances (both legal and illegal).

### Limitations

Data for the present work was drawn from a routine assessment whose primary focus was not on suicide research. A strength of this procedure is that it gives insight into the reality of clinical practice, all patients treated in routine clinical care were eligible and represented in the data (precluding selection biases), and the available data was very comprehensive. A downside, on the other hand, is the lack of more in-depth information about patients’ suicide attempts: there was no information about methods or intent and we do not know how long ago the reported attempts were, all of which present limitations. Furthermore, as most of the information was assessed via self-report, we must consider memory biases, lapses or denial. Since suicidality is highly stigmatized it is conceivable that patients may not want to disclose previous attempts, especially when they are newly admitted and still building trust with healthcare professionals they do not yet know. Disclosure might also have had negative implications in the past^58^. However, empirical evidence indicates that inconsistencies in the reporting of STBs are unlikely due to motivated concealment^59^. Further, there was no information about non-suicidal self-injury, which is an important risk indicator as repeated self-harm may lead to an increased tolerance for self-inflicted pain, making the transition to suicidal behavior more feasible^60^. Other information of interest would have been patients' income/better insight into their overall socioeconomic status.

In addition, we were only able to relate patients' current characteristics to their past behavior instead of investigating the occurrence of suicide attempts prospectively. While some of the investigated variables can be assumed to be stable over years or decades (such as gender, level of education, or personality), others could show great fluctuations (such as symptom burden). While future research should address the question of how these characteristics are related to suicide attempts that will happen in the future, including after an inpatient stay, the present results are still informative as they yield a (previously lacking) comprehensive investigation of the specific vulnerabilities of inpatients who made suicide attempts in the past. Furthermore, past attempts have implications for patients' future well-being and suicide risk, not only because they can be experienced as traumatic^61^, but also because they confer elevated acquired capability for suicide^62^.

## Conclusion

In a large, naturalistic patient sample, there was a substantial proportion of individuals reporting a lifetime a history of suicide attempts. They suffered from comparatively higher symptom burden than other patients and while they did not differ from them in all respects that were investigated, some differences mapped on to previously identified risk factors for suicidal behavior. Hence, observations from clinical practice support conceptual models such as the IMV model that distinguishes risk factors for suicidal ideation and suicidal behavior. Such theories can also be used to guide assessments, which, in turn, can yield information about crucial factors for screening and targeted interventions in clinical practice. While some of the identified differences are stable or cannot be altered because they are in the past (such as childhood abuse and neglect), others, such as the level of personality functioning, have implications for treatment planning, and also give an insight into the particular challenges individuals with a history of suicide attempts face in daily life.

## Data Availability

The data are confidential and cannot be posted publicly. An anonymized version of the original dataset and analysis code supporting the findings of the study is available upon reasonable request to the corresponding author.
